# Arachidonic acid and calcium metabolism in rnelittin stimulated neutrophils

**DOI:** 10.1155/S0962935192000462

**Published:** 1992

**Authors:** Ole H. Nielsen, Pierre N. Bouchelouche, Dag Berild

**Affiliations:** 1 Department of Medical Gastroenterology C Herlev Hospital University of Copenhagen Herlev Ringvej, Herlev DK-2730 Denmark; 2 Department of Clinical Chemistry Herlev Hospital University of Copenhagen Herlev Ringvej, Herlev DK-2730 Denmark

## Abstract

Melittin, the predominant fraction of bee venom proteins, was studied in an experimental model of human neutrophil granulocytes to reveal its influence on eicosanoid release, metabolism and receptor function in relation to intracellular calcium metabolism. Melittin (2 μmol/l) was as potent as the calcium ionophore A23187 (10 μmol/l) for activation of 5-lipoxygenase, releasing arachidonate only from phosphatidyl-choline and phosphatidyl-ethanolamine of cellular membranes, as judged from the decreases in radioactivity by 15.4% and 30.5%, respectively. The mechanism responsible for the release of arachidonate from cellular membranes is closely coupled to cellular calcium metabolism, and melittin was found to promote calcium entry through receptor gated calcium channels, probably due to an activation of phospholipase A_2_. Furthermore, a down-regulation of leukotriene B_4_ receptors was seen. The maximal number of binding sites per cell was reduced from a median of 1520 to 950 with melittin (1 μmol/l). The study has revealed some factors important for the inflammatory mechanisms mediated by melittin.


Mediators of Inflammation, 1, 313–317 (1992)

### Arachidonic acid and calcium metabolism in melittin stimulated neutrophils

Ole H. Nielsen,[1,CA] Pierre N. Bouchelouche[2] and Dag Berild[1]

[1]Department of Medical Gastroenterology C; and [2]Department of Clinical Chemistry, Herlev Hospital, University of Copenhagen, Herlev Ringvej, DK-2730 Herlev, Denmark

[CA] Corresponding Author

**MELITTIN, the predominant fraction of bee venom proteins, was studied in an experimental model of human neutrophil granulocytes to reveal its influence on eicosanoid release, metabolism and receptor function in relation to intracellular calcium metabolism. Melittin (2 μmol/l) was as potent as the calcium ionophore A23187 (10 μmol/l) for activation of 5-lipoxygenase, releasing arachidonate only from phosphatidyl-choline and phosphatidyl-ethanolamine of cellular membranes, as judged from the decreases in radioactivity by 15.4% and 30.5%, respectively. The mechanism responsible for the release of arachidonate from cellular membranes is closely coupled to cellular calcium metabolism, and melittin was found to promote calcium entry through receptor gated calcium channels, probably due to an activation of phospholipase $A_2$. Furthermore, a down-regulation of leukotriene $B_4$ receptors was seen. The maximal number of binding sites per cell was reduced from a median of 1520 to 950 with melittin (1 μ mol/l). The study has revealed some factors important for the inflammatory mechanisms mediated by melittin.**

**Key words:** Arachidonic acid, Calcium, Cytosol, Ionophores, Leukotrienes, Melittin, Neutrophils, *N*-formylmethionine-leucyl-phenylalanine, Phospholipids, Receptors

### Introduction

Melittin is a polypeptide toxin which constitutes more than 50% of bee venom proteins.[1] It has been claimed to release endogenous arachidonic acid (AA) from cultured cells and to increase formation of prostanoids through activation of membrane bound enzymes.[2–4] Further, melittin was found to be able to stimulate exogenous non-incorporated AA metabolism in human polymorphonuclear neutrophils (PMN),[5] and recent research has dealt with the interaction between melittin and cellular membranes.[6]

The aims of the present work were: (1) to assess if melittin was a stimulator of endogenous AA metabolism in purified human PMNs; (2) to compare its potency with that of the calcium ionophore A23187; (3) to reveal where in the phospholipid pool AA was mobilized by melittin challenge; (4) to evaluate its influence on cellular calcium metabolism; and finally (5) to investigate its possible action on surface leukotriene $B_4$ receptors.

### Materials and Methods

In six experiments neutrophils were isolated from EDTA-blood (0.2 mmol/l), with a recovery of 45%, and a purity of more than 95%, by: (1) methylcellulose (0.8%) sedimentation of erythrocytes; (2) washing and gradient centrifugation of 'buffy coat' leukocytes according to Böyum;[7] and finally (3) hypotonic lysis of residual erythrocytes. Incorporation of 1-$^{14}$C-AA (37 × $10^3$ Bq/ml, 2.2 × $10^9$ Bq/mmol) (Amersham International, UK) with labelling of intracellular pools of phospholipids proceeded for 5 h at 37°C under 5% carbon dioxide and 95% atmospheric air in RPMI 1640 (5 × $10^6$ cells) (5% autologous serum).[8] After removal of excess extracellular AA by washing, the cells were challenged with melittin (0.05–10 μmol/l) for various lengths of time (Sigma Chemical Co., St Louis, MO, USA) or calcium ionophore A23187 (Calbiochem, La Jolla, CA, USA) (10 μmol/l) for 15 min, which was found to be optimal from previous experiments.[8] Released eicosanoids were extracted with dichloromethane:methanol, 2:1; separated by thin layer chromatography, developing solvents 1: (supernatants), chloroform: methanol: acetic acid: water, 90:9:1:0.65; 2: (total cell suspensions), dichloromethane:methanol: 2-propanol, 0.25% KCl:ethylacetate, 30:9:25:6:18; and quantified by autoradiography and laser densitometry.[8]

Identification of the radioactive spots was performed by co-chromatography with pure standards of phospholipids (Sigma Chemical Co.), 5-hydroxyeicosatetraenoic acid (5-HETE), leukotriene $B_4$ ($LTB_4$) (Paesel Gmbh, Frankfurt am Main, Germany, and 12-hydroxy-heptadecatrienoic acid (HHT) (Upjohn Company, Kalamazoo, MI, USA). Further identification was performed by high-

© 1992 Rapid Communications of Oxford Ltd performance liquid chromatography (HPLC) as earlier described.[8] The intra-assay coefficient of variation for release of AA metabolites was approximately 15%.[8]

The viability of the PMNs were assessed by the trypan blue exclusion technique. In six separate experiments PMNs ($5 \times 10^6$/ml) were incubated with Fura-2/am (Sigma Chemical Co.) (2 μmol/l) for 15 min. The cells were centrifuged, washed twice, and resuspended. Fluorescence was recorded with a Hitachi 4000 fluorescence spectrophotometer. For the calibration of Fura-2 fluorescence as a function of $(Ca^{2+})_i$, we used digitonin (10 mg/ml) to obtain a maximum fluorescence signal, $F_{max}$, followed by the addition of EGTA for determination of $F_{min}$. Intermediate values for $Ca^{2+}$ corresponding to an intracellular Fura-2 fluorescence, F, were calculated by the equation: $Ca^{2+} = Kd\ (F - F_{min}/F_{max} - F)$, assuming an effective Kd of 224 nmol/l.[9] *N*-formylmethionine-leucyl-phenylalanine (fMLP) (Sigma Chemical Co.) was used in the concentration range $10 \times 10^{-9}-10^{-6}$ mol/l.

In separate receptor studies duplicate suspensions of PMNs ($10^7$/ml) were incubated with radioactive $^3$H-LTB$_4$ (specific activity 6.3–8.5 × $10^3$ GBq/mmol, Amersham International, UK), 0.1 nmol/l–2.5 nmol/l at 4°C for 60 min. Following incubation, the cells were rapidly centrifuged through a precooled oil phase.[9] The tips of the tubes were cut off, and cell bound radioactivity was determined in a tracer analytical scintillation counter with an automatic quench correction. Nonspecific binding was determined by adding a 1000-fold excess of non-radioactive LTB$_4$ (Paesel Gmbh, Germany).[10]

In specific experiments, 1 μmol/l melittin was added to the cell suspensions at 37°C for 5 min. The cells were then rapidly cooled to 4°C and binding experiments were done. For estimation of the dissociation constant (Kd) and receptor number per cell ($B_{max}$) a Scatchard plot was applied.[11]

### Results

*Potential sources of arachidonic acid:* Optimal conditions for AA release and metabolism were 2 μmol/l melittin (Table 1) for 10 min (Fig. 1), which resulted in the median release of 415 Bq/5 × $10^6$ PMNs (range 289–618) compared to 670 Bq/5 × $10^6$ PMNs (range 260–990) for A23187 ($p < 0.05$).

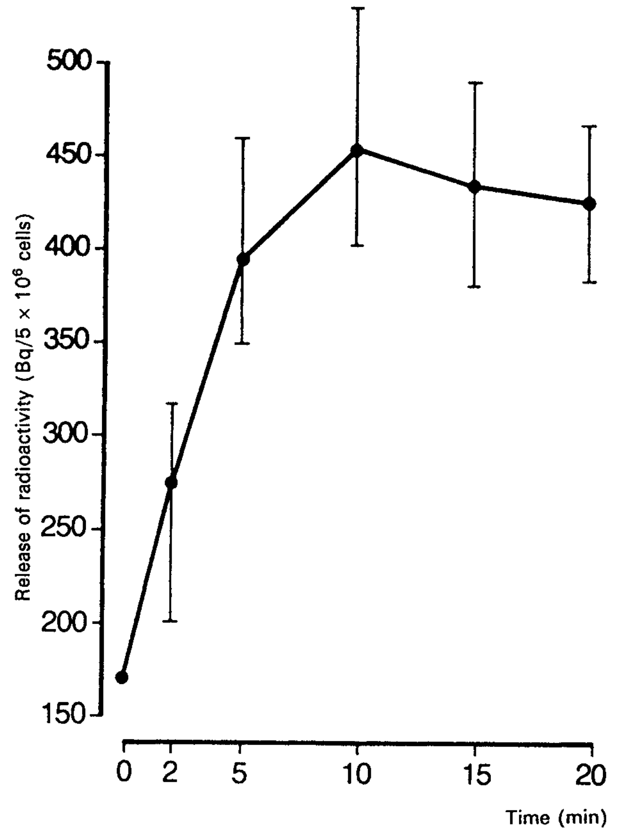

FIG. 1. Time course experiments for stimulation of 1-$^{14}$C-arachidonic acid labelled neutrophils with melittin (2 μmol/l). Values are given as medians with ranges for six experiments.

The radioactivity, when challenged with melittin (2 μmol/l, 10 min) or A23187 (10 μmol/l, 15 min), was distributed on eicosanoids and unmetabolized AA, as seen in Table 2. No significant differences occurred.

The substrate for formation of most of the AA following melittin stimulation was mobilized from phosphatidyl-choline (PC) and phosphatidyl-ethanolamine (PE), owing to the relative decreases in median radioactivity by 15.4% ($p < 0.01$), and 30.5% ($p < 0.01$), respectively (Fig. 2). No significant changes were found for phosphatidyl-inositol (PI) or phosphatidyl-serine (PS).

*Calcium mobilization:* Addition of melittin to PMNs caused a dose-related increase in intracellular $Ca^{2+}$ $(Ca^{2+})_i$ concentrations (Fig. 3). This rise was

**Table 1.** Dose response for release of radioactivity (eicosanoids and unmetabolized arachidonic acid) by melittin (15 min stimulation period) ($n = 6$). Values for optimal stimulation was taken to 100% (equal to 415 Bq/5 × $10^6$ PMNs). Medians with 95% confidence limits on medians are given

| Concentration of melittin (μmol/l) | 0.05 | 0.1 | 0.5 | 1 | 2 | 5 | 10 |
|---|---|---|---|---|---|---|---|
| Radioactivity | 46<br>(41–49) | 48<br>(46–51) | 52<br>(49–55) | 77<br>(72–80) | 100<br>(93–107) | 68<br>(64–71) | 74<br>(69–77) |

**Table 2.** Percentage distribution of radioactivity on eicosanoids and unmetabolized arachidonic acid after stimulation with melittin (2 μmol/l, 10 min) or A23187 (10 μmol/l, 15 min). ($n = 6$). Medians and ranges are given

| | Arachidonic acid | 5-HETE | $LTB_4$ | HHT |
|---|---|---|---|---|
| Melittin | 68.1 | 13.5 | 5.8 | 1.7 |
| | (63.7–76.7) | (11.3–14.7) | (1.5–6.8) | (1.4–2.4) |
| A23187 | 67.0 | 14.9 | 5.3 | 2.2 |
| | (56.2–80.4) | (9.5–19.4) | (2.8–9.6) | (1.0–5.3) |

abolished in $Ca^{2+}$-free media, suggesting that a melittin-induced increase in $(Ca^{2+})_i$) was mediated by an increase of the plasma membrane permeability to $Ca^{2+}$.

The characteristics of the calcium signal (time courses of changes in $(Ca^{2+})_i$) generated upon activation of phospholipase $A_2$ ($PLA_2$):(melittin) and phospholipase C (PLC):($LTB_4$ and fMLP) of human PMNs are shown in Fig. 4. As can be seen from this figure the $Ca^{2+}$ response pattern is similar after addition of $LTB_4$ or fMLP. This response is transient, as the rise in $(Ca^{2+})_i$ in the bulk cytosol is due to a release of $Ca^{2+}$ from intracellular stores. With melittin, however, the

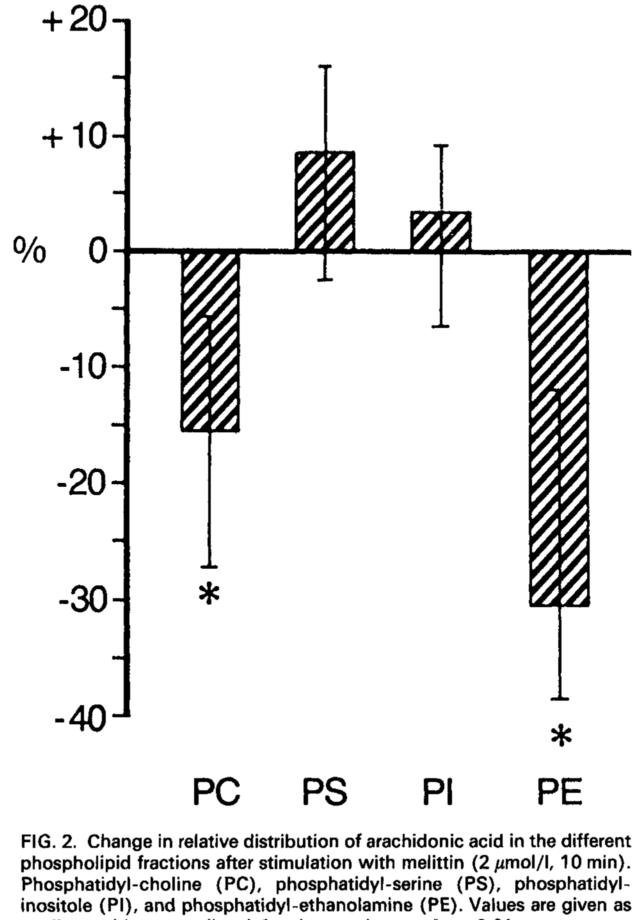

FIG. 2. Change in relative distribution of arachidonic acid in the different phospholipid fractions after stimulation with melittin (2 μmol/l, 10 min). Phosphatidyl-choline (PC), phosphatidyl-serine (PS), phosphatidyl-inositole (PI), and phosphatidyl-ethanolamine (PE). Values are given as medians with ranges (bars) for six experiments. *$p < 0.01$.

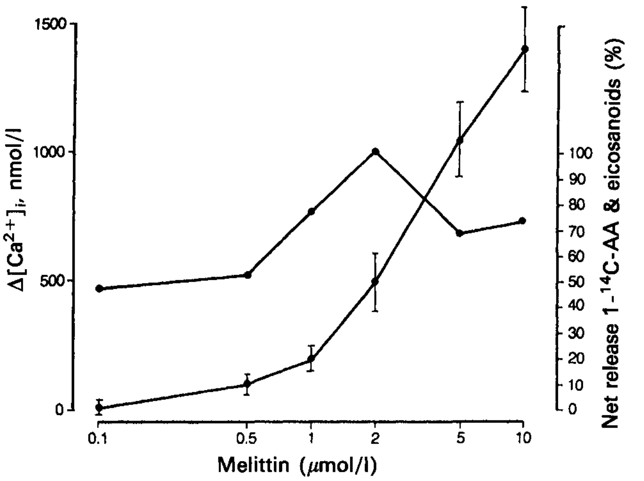

FIG. 3. Release of radioactivity (eicosanoids and unmetabolized arachidonic acid) ●—● (median values) and change in intracellular free $Ca^{2+}$ [$(Ca^{2+})_i$] ●—● (median values and ranges) in human neutrophil granulocytes by the stimulation with melittin.

$Ca^{2+}$ rise shows a sustained phase which is due to influx of $Ca^{2+}$ across the plasma membrane, and the effect is sustained as long as melittin is present in the medium. Validation of these results is supported by the experiments performed in a $Ca^{2+}$-free medium (Fig. 5). When extracellular $Ca^{2+}$ was removed, the melittin-induced $Ca^{2+}$-influx was inhibited (Fig. 5). However, in a $Ca^{2+}$-free medium, $LTB_4$ and fMLP still result in a transient rise in free $(Ca^{2+})_i$ (data not shown).

However, in a $Ca^{2+}$-free medium, if melittin is added before $LTB_4$, the increase in cytosolic free calcium elicited by $LTB_4$ is completely inhibited (Fig. 5). To obtain a rise in cytosolic free $Ca^{2+}$, a higher concentration (50-fold) of $LTB_4$ is needed. Further, the $Ca^{2+}$ rise ($Ca^{2+}$ release from

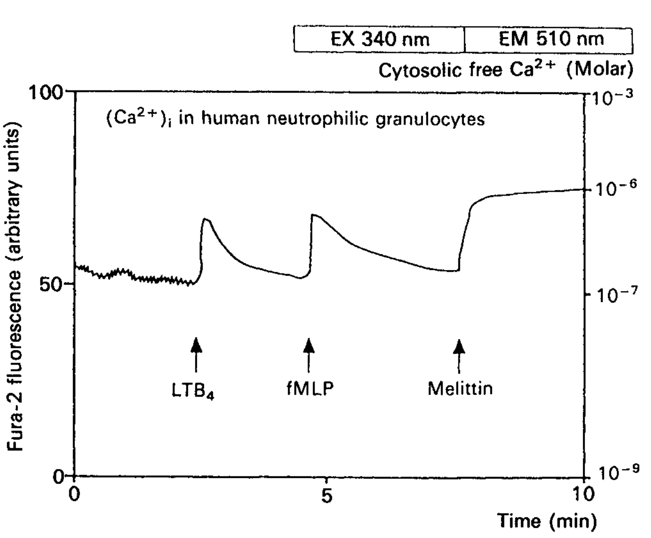

FIG. 4. Time-course changes in fura-2 fluorescence and $(Ca^{2+})_i$ in human PMNs following addition of $LTB_4$ ($3 \times 10^{-10}$ mol/l), fMLP ($0.1 \times 10^{-6}$ mol/l), and melittin ($3.5 \times 10^{-6}$ mol/l). Typical trace of five experiments.

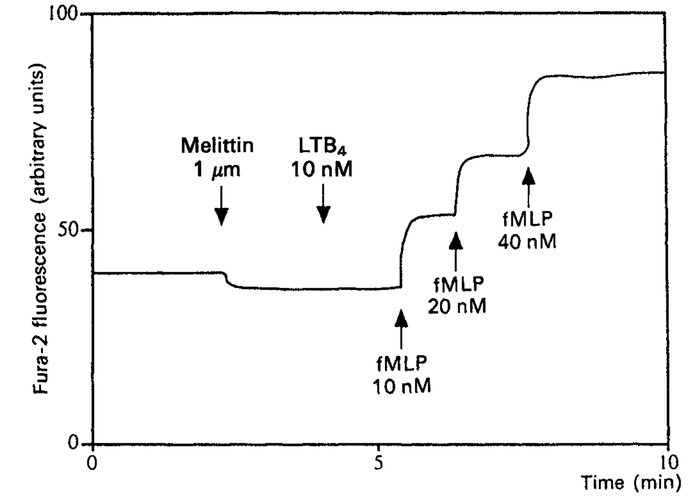

FIG. 5. Influence of melittin (1 μmol/l) on $(Ca^{2+})_i$ after stimulation of human PMNs with $LTB_4$ (10 nmol/l) or fMLP (10–40 nmol/l) in calcium free medium.

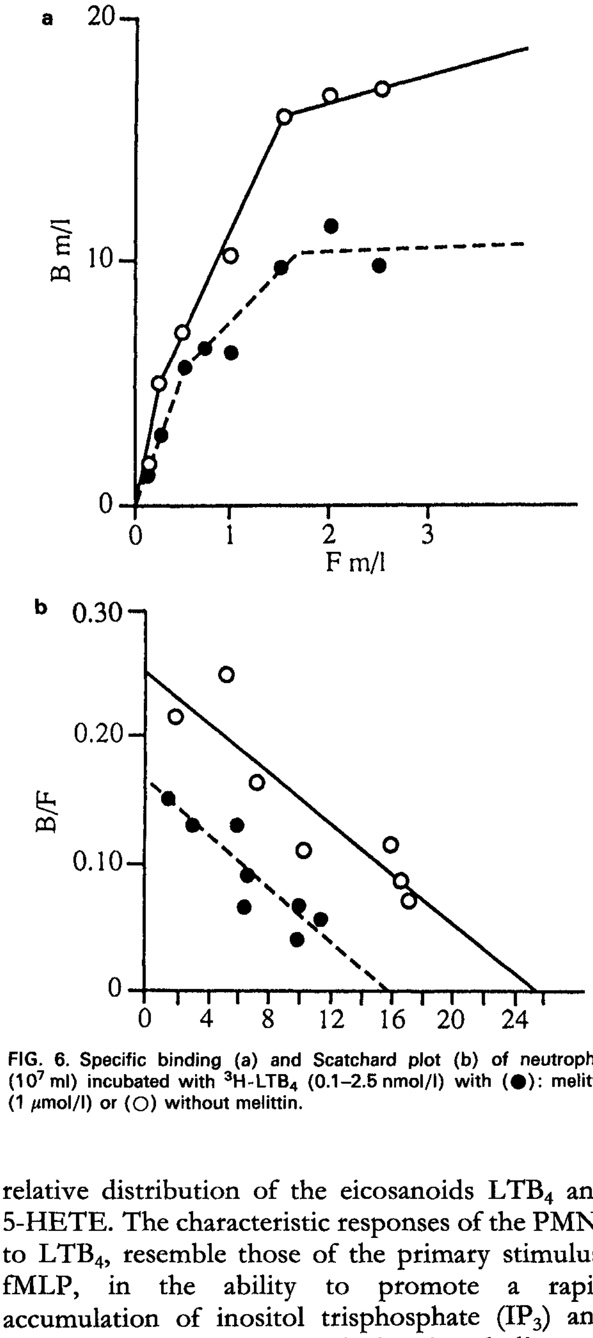

FIG. 6. Specific binding (a) and Scatchard plot (b) of neutrophils ($10^7$ ml) incubated with $^3H$-$LTB_4$ (0.1–2.5 nmol/l) with (●): melittin (1 μmol/l) or (○) without melittin.

intracellular stores) induced by fMLP is unaffected by melittin.

To investigate whether inhibition of the $Ca^{2+}$ signal by $LTB_4$ paralleled a decrease in the number or affinity of $LTB_4$ receptors, receptor ligand binding assay experiments were performed.

Figure 6 demonstrates specific binding (a) of $LTB_4$ and the corresponding Scatchard plot, and (b) when PMNs were incubated with $^3H$-$LTB_4$ 0.1–2.5 nmol/l. The nonspecific binding was linear with increasing ligand concentration. From the Scatchard plot it can be derived that the Kd is approximately the same, 0.95 nmol/l and 1.05 nmol/l in cell suspensions with and without melittin (1 μmol/l), respectively. However, melittin significantly reduced the maximal number of $LTB_4$ binding sites per cell ($B_{max}$) from 1520 to 950 under identical experimental conditions.

The viability of the PMNs after challenge with melittin or A23187 in the concentration ranges used was more than 97% as assessed by the trypan blue exclusion technique.

### Discussion

The data demonstrate that the bee venom polypeptide, melittin, is a potent stimulator of endogenous AA metabolism to mono- and di-hydroxy products, including $LTB_4$, in human PMNs which are of importance for inflammatory reactions.[12,13] Its potency regarding phospholipases stimulation in human PMNs is about 2/3 that of A23187, which is assumed to produce a maximal stimulation of phospholipases and synthesis of leukotrienes in response to calcium influx. However, regarding 5-lipoxygenase stimulation, an enzyme which has been shown to exhibit an absolute requirement of calcium ions,[14] melittin and A23187 were equipotent, as evaluated from the relative distribution of the eicosanoids $LTB_4$ and 5-HETE. The characteristic responses of the PMNs to $LTB_4$, resemble those of the primary stimulus, fMLP, in the ability to promote a rapid accumulation of inositol trisphosphate ($IP_3$) and calcium mobilization through the phospholipase C system.[15–17] Further, melittin stimulates cyclooxygenase, as evaluated from the production of HHT, with a similar potency as found for A23187.

Stimulation of $PLA_2$ as well as PLC, which are essential for AA metabolism and which have been demonstrated in PMN membranes,[18] appears to involve cellular calium.[19] Increase in $(Ca^{2+})_i$ could be effected either by PLC mediated $IP_3$ accumulation, or by $Ca^2$ influx through receptor operated (voltage independent) $Ca^{2+}$ channels. If calcium dependent phospholipases are regulators of AA release, then agents that stimulate $PLA_2$ might be expected to increase calcium availability. Melittin has earlier been described to stimulate phospholipase $A_2$ in human leukocytes in the presence of exogenous AA,[5] and an ability to alter membrane permeability to calcium has been further suggested.[5] The calcium antagonists, verapamil and nifedipine, at concentrations known to block classical voltage dependent calcium channels, had no effect on the $Ca^{2+}$ influx induced by melittin, possibly due to receptor operated $Ca^{2+}$ influx.[9] This is contrary to apamin, another toxin from bee venom, which affects the calcium channel function.[6] The possibility that melittin interferes with calcium influx via secondary messengers (i.e. $IP_3$) is out of question, since there was no increase in $(Ca^{2+})_i$ in calcium-free media. Further, melittin only released AA from PC and PE and not via the classical way, which involves PI, PE, PC as the three sources of AA.[19] It has been described earlier, that AA is mobilized from PC, PE, and PI using the calcium ionophore A23187.[8]

The present investigation has demonstrated that melittin increases the plasma permeability to calcium, and that cytosolic free $Ca^{2+}$ is closely coupled to melittin induced activation of $PLA_2$. Further, melittin affects PMN surface $LTB_4$ receptors, as it significantly reduces the maximal number of $LTB_4$ binding sites per cell ($B_{max}$). One possible explanation is that melittin generates $LTB_4$ from PMNs.[5] $LTB_4$ is then exported to the extracellular medium where it subsequently acts as a receptor agonist on the PMN surfaces with a resulting down-regulation of $LTB_4$ receptors. Another explanation is that activators of protein kinase C (PKC), an important component of the signal transduction pathway in human PMNs, cause cells to become unresponsive to $LTB_4$, but not to fMLP.[20] However, in this respect melittin has earlier been found to be a PKC inhibitor.[21] Therefore melittin may act indirectly as an activator of PKC through AA release.[22] The latter (AA) is known to induce diacylglycerol (DAG) generation, which then activates PKC.[23] Since the production of AA is high following addition of melittin, we believe that bee venom via DAG is an indirect PKC-activator. This could explain the deactivation and the decrease in the expression of $LTB_4$ receptors found in the present study, as explained above.[20,23]

In conclusion, melittin is a potent challenger of 5-lipoxygenase AA metabolism in human PMNs. Its potency regarding phospholipases stimulation is about 2/3 that of A23187 which is assumed to produce a maximal stimulation in response to calcium influx. Further, melittin mobilizes AA from PC and PE, whereas PI and PS seem to be unaffected, and melittin promotes $Ca^{2+}$ entry through receptor gated $Ca^{2+}$-channels, probably due to a direct activation of phospholipase $A_2$. Receptor studies indicate that melittin further affects the total number of $LTB_4$-receptors, either by a down-regulatory mechanism or via the PKC system. The present data provide evidence for the complicated mechanism of melittin, and its sensitivity to arachidonate metabolism, cellular eicosanoid receptors and intracellular calcium suggests that these factors may play a role for the inflammation mediated by melittin.

### References

1. Habermann E. Bee and wasp venoms. *Science* 1972; **177**: 314–322.
2. Shier WT. Activation of high levels of endogenous phospholipase $A_2$ in cultured cells. *Proc Natl Acad Sci USA* 1979; **76**: 195–199.
3. Hojvat SA, Musch MW, Miller RJ. Stimulation of prostaglandin production in rabbit ileal mucosa by bradykinin. *J Pharmacol Exp Ther* 1983; **226**: 749–755.
4. Mauer P, Moskowitz MA, Levine L, Melamed E. The synthesis of prostaglandins by bovine cerebral microvessels. *Prostaglandins Med* 1980; **4**: 153–161.
5. Salari H, Braquet P, Borgeat P. Stimulation of lipoxygenase product synthesis in human leukocytes and platelets by melittin. *Mol Pharmacol* 1985; **28**: 546–548.
6. Hamilton SL, Perez M. Toxins that affect voltage-dependent calcium channels. *Biochem Pharmacol* 1987; **36**: 3325–3329.
7. Böyum A. Isolation of leucocytes from human blood. *Scand J Clin Lab Invest* 1968; **21** (suppl 97): 9–29.
8. Nielsen OH, Bukhave K, Ahnfelt-Rønne I, Elmgreen J. Arachidonic acid metabolism in human neutrophils: lack of effect of cyclosporine A. *Int J Immunopharmacol* 1986; **8**: 419–426.
9. Bouchelouche PN, Hainau B, Frederiksen O. Effect of BAY K8644 on cytosolic free calcium in isolated rabbit gall-bladder epithelial cells. *Cell Calcium* 1989; **10**: 37–46.
10. Bouchelouche PN, Berild D. Possible existence of leukotriene $D_4$ receptors and mechanism of their signal transduction in human polymorphonuclear leukocytes. *Scand J Clin Lab Invest* 1991; **51** (suppl 204): 47–55.
11. Scatchard G. The attractions of proteins for small molecules and ions. *Ann NY Acad Sci* 1949; **51**: 660–672.
12. Nielsen OH. *In vitro* studies on the significance of arachidonate metabolism and other oxidative processes in the inflammatory response of human neutrophils and macrophages—with special reference to chronic inflammatory bowel disease. *Scand J Gastroenterol* 1988; **23** (suppl 150): 1–21.
13. Naccache PH, Sha'afi RI. Arachidonic acid, leukotriene $B_4$, and neutrophil activation. *Ann NY Acad Sci* 1983; **414**: 125–139.
14. Rouzer CA, Shimizu T, Samuelsson B. On the nature of the 5-lipoxygenase reaction in human leukocytes: characterization of a membrane associated stimulatory factor. *Proc Natl Acad Sci USA* 1985; **82**: 7505–7509.
15. Bradford PG, Rubin RP. Characterization of formylmethionyl-leucyl-phenylalanine stimulation of inositol trisphosphate accumulation in rabbit neutrophils. *Mol Pharmacol* 1985; **27**: 74–78.
16. Goldman DW, Grifford LA, Olson DM, Goetzl EJ. Transduction by leukotriene $B_4$ receptors of increases in cytosolic calcium in human polymorphonuclear leukocytes. *J Immunol* 1985; **135**: 525–530.
17. Bouchelouche PN, Ahnfelt-Rønne I, Thomsen MK. $LTD_4$ increases cytosolic free calcium and inositol phosphates in human neutrophils: inhibition by the novel $LTD_4$ receptor antagonist, SR2640, and possible relation to modulation of chemotaxis. *Agents Actions* 1990; **29**: 299–307.
18. Victor M, Weiss J, Klempner MS, Elsbach P. Phospholipase $A_2$ activity in the plasma membrane of the human polymorphonuclear leukocytes. *FEBS Lett* 1981; **136**: 298–300.
19. Rubin RP. Calcium-phosphatidylinositol interactions in secretory cells and the role of arachidonic acid. In: Bleasdale JE, Eichberg J, Hauser G, eds. *Inositol and Phosphoinositides: Metabolism and Regulation.* Clifton, NJ: Humana Press, 1985; 367–387.
20. Goldman DW. Regulation of the receptor system for leukotriene $B_4$ on human neutrophils. *Ann NY Acad Sci* 1988; **524**: 187–195.
21. Katoh N, Raynor RL, Wise BC, *et al.* Inhibition by melittin of phospholipid-sensitive and calmodulin-sensitive $Ca^{2+}$ dependent protein kinases. *Biochem J* 1982; **202**: 217–224.
22. McPhail LC, Clayton CC, Snyderman R. A potential second messenger role for unsaturated fatty acids: activation of calcium-dependent protein kinases. *Science* 1984; **224**: 622–624.
23. Turner RS, Kuo JF. Phospholipid-sensitive $Ca^{2+}$-dependent protein kinase (protein kinase C): the enzyme, substrates, and regulation. In: Kuo JF, ed. *Phospholipids and Cellular Regulation, Volume II.* Boca Raton, FL: CRC Press, Inc., 1985; 90–95.

ACKNOWLEDGEMENTS. The authors are grateful to Birgit Dejbjerg, Helma Furhauge, and Hanne Kargaard for excellent technical assistance. This work was supported by handelsgartner Ove Villiam Buhl Olesen's and aegtefaelle Edith Buhl Olesen's Foundation, Jacob Madsen's and hustru Olga Madsen's Foundation, and the Danish Medical Research Council.

**Received 4 July 1992; accepted in revised form 24 July 1992**

## References

[PDF_00484] Bouchelouche P. N., Ahnfelt-Rønne I., Thomsen M. K. (1990). LTD4 increases cytosolic free calcium and inositol phosphates in human neutrophils: inhibition by the novel LTD4 receptor antagonist, SR2640, and possible relation to modulation of chemotaxis.. Agents Actions.

[PDF_00464] Bouchelouche P. N., Berild D. (1991). Possible existence of leukotriene D4 receptors and mechanism of their signal transduction in human polymorphonuclear leukocytes.. Scand J Clin Lab Invest Suppl.

[PDF_00461] Bouchelouche P. N., Hainau B., Frederiksen O. (1989). Effect of BAY K 8644 on cytosolic free calcium in isolated rabbit gall-bladder epithelial cells.. Cell Calcium.

[PDF_00478] Bradford P. G., Rubin R. P. (1985). Characterization of formylmethionyl-leucyl-phenylalanine stimulation of inositol trisphosphate accumulation in rabbit neutrophils.. Mol Pharmacol.

[PDF_00481] Goldman D. W., Gifford L. A., Olson D. M., Goetzl E. J. (1985). Transduction by leukotriene B4 receptors of increases in cytosolic calcium in human polymorphonuclear leukocytes.. J Immunol.

[PDF_00495] Goldman D. W. (1988). Regulation of the receptor system for leukotriene B4 on human neutrophils.. Ann N Y Acad Sci.

[PDF_00442] Habermann E. (1972). Bee and wasp venoms.. Science.

[PDF_00454] Hamilton S. L., Perez M. (1987). Toxins that affect voltage-dependent calcium channels.. Biochem Pharmacol.

[PDF_00445] Hojvat S. A., Musch M. W., Miller R. J. (1983). Stimulation of prostaglandin production in rabbit ileal mucosa by bradykinin.. J Pharmacol Exp Ther.

[PDF_00497] Katoh N., Raynor R. L., Wise B. C., Schatzman R. C., Turner R. S., Helfman D. M., Fain J. N., Kuo J. F. (1982). Inhibition by melittin of phospholipid-sensitive and calmodulin-sensitive Ca2+-dependent protein kinases.. Biochem J.

[PDF_00448] Maurer P., Moskowitz M. A., Levine L., Melamed E. (1980). The synthesis of prostaglandins by bovine cerebral microvessels.. Prostaglandins Med.

[PDF_00500] McPhail L. C., Clayton C. C., Snyderman R. (1984). A potential second messenger role for unsaturated fatty acids: activation of Ca2+-dependent protein kinase.. Science.

[PDF_00473] Naccache P. H., Sha'afi R. I. (1983). Arachidonic acid, leukotriene B4, and neutrophil activation.. Ann N Y Acad Sci.

[PDF_00458] Nielsen O. H., Bukhave K., Ahnfelt-Rønne I., Elmgreen J. (1986). Arachidonic acid metabolism in human neutrophils: lack of effect of cyclosporine A.. Int J Immunopharmacol.

[PDF_00469] Nielsen O. H. (1988). In vitro studies on the significance of arachidonate metabolism and other oxidative processes in the inflammatory response of human neutrophils and macrophages. With special reference to chronic inflammatory bowel disease.. Scand J Gastroenterol Suppl.

[PDF_00475] Rouzer C. A., Shimizu T., Samuelsson B. (1985). On the nature of the 5-lipoxygenase reaction in human leukocytes: characterization of a membrane-associated stimulatory factor.. Proc Natl Acad Sci U S A.

[PDF_00451] Salari H., Braquet P., Borgeat P. (1985). Stimulation of lipoxygenase product synthesis in human leukocytes and platelets by melittin.. Mol Pharmacol.

[PDF_00443] Shier W. T. (1979). Activation of high levels of endogenous phospholipase A2 in cultured cells.. Proc Natl Acad Sci U S A.

[PDF_00488] Victor M., Weiss J., Klempner M. S., Elsbach P. (1981). Phospholipase A2 activity in the plasma membrane of human polymorphonuclear leukocytes.. FEBS Lett.

